# ﻿Review of Orchidaceae of the northern part of Kazakhstan

**DOI:** 10.3897/phytokeys.229.105457

**Published:** 2023-07-27

**Authors:** Serik A. Kubentayev, Petr G. Efimov, Daniyar T. Alibekov, Andrey N. Kupriyanov, Klara S. Izbastina, Aizhan E. Khalymbetova, Yuri V. Perezhogin

**Affiliations:** 1 Astana Botanical Garden, 16 Orynbor Str., 010016, Astana, Kazakhstan Astana Botanical Garden Astana Kazakhstan; 2 Komarov Botanical Institute of the Russian Academy of Sciences, 2 Professor Popov Str., 197022, Saint-Petersburg, Russia Komarov Botanical Institute of the Russian Academy of Sciences Saint-Petersburg Russia; 3 Federal Research Center of Coal and Coal Chemistry of Siberian Branch of the Russian Academy of Sciences, 18 Sovetsky Ave., 650000, Kemerovo, Russia Federal Research Center of Coal and Coal Chemistry of Siberian Branch of the Russian Academy of Sciences Kemerovo Russia; 4 S. Seifullin Kazakh Agrotechnical Research University, 62 Zhengis Ave., 010000, Astana, Kazakhstan S. Seifullin Kazakh Agrotechnical Research University Astana Kazakhstan; 5 L.N. Gumilyov Eurasian National University, 2 Satpayev Str., 010000, Astana, Kazakhstan L.N. Gumilyov Eurasian National University Astana Kazakhstan; 6 A. Baitursynov Kostanay Regional University, 47 Baytursynov Str., 110000, Kostanay, Kazakhstan A. Baitursynov Kostanay Regional University Kostanay Kazakhstan

**Keywords:** Biodiversity, conservation status, distribution, flora of Kazakhstan, orchid hotspot, rare plants

## Abstract

We present a review of Orchidaceae Juss. of the northern part of Kazakhstan, within the steppe, forest-steppe and semi-desert habitats of the country (Pavlodar, northern Kazakhstan, Kostanay, Akmola, Aktobe, West Kazakhstan, partially Karaganda and East Kazakhstan regions). The investigation is based on herbarium materials, literature data and field observations. We examined material from the following herbarium collections: LE, MW, TK, MHA, SVER, KUZ, ALTB, AA, NUR, KG, KSPI, NS, NSK, MOSP, ORIS, PPIU, totalling 288 herbarium specimens. The paper presents data in the form of revision, focusing on orchids of the northern part of Kazakhstan. It is accompanied by maps indicating localities, notes on habitat preferences, phenology and conservation status. A total of 25 species of 16 genera were recorded, of which eight are included in the Red Book of Kazakhstan (2014). According to our data, we propose to enlarge the number of protected orchids by adding the following nine species: *Corallorhizatrifida*, *Epipactisatrorubens*, *Gymnadeniaconopsea*, *Hammarbyapaludosa*, *Herminiummonorchis*, *Liparisloeselii*, *Malaxismonophyllos*, *Neottiacamtschatea* and *Spiranthesaustralis*. The most widespread species in the studied region are *Dactylorhizaincarnata*, *D.umbrosa* and *Epipactispalustris*. The rarest species (single locality only) are *Epipactisatrorubens*, *E.helleborine*, *Epipogiumaphyllum*, *Hammarbyapaludosa* and *Herminiummonorchis*.

## ﻿Introduction

Orchids are one of the largest families in the world, numbering, according to various data, from 28,000 to 30,500 species (Chase 2005; Chase et al. 2015; Christenhusz and Byng 2016; Hassler 2023). Due to human encroachment and climate change, as well as other factors, many orchid species are at risk of extinction (Fay 2018; Zizka et al. 2021). Eight species are listed in the Red Book of Kazakhstan (2014).

The diversity of Orchidaceae Juss. in Kazakhstan is low due to the prevalence of an arid climate with a rather harsh temperature regime in the cold period. According to the last vascular plant list of Kazakhstan by Abdulina (1999), there are 31 species of orchids from 18 genera in Kazakhstan. However, several recent additions prove that the diversity of orchids in Kazakhstan is insufficiently studied. The following taxa were discovered in Kazakhstan for the first time since 1999: Cypripedium×ventricosum Sw. (Kotuhov et al. 2009, 2018), *Epipactisatrorubens* (Hoffm.) Besser (Perezhogin et al. 2015), *Hammarbyapaludosa* (L.) Kuntze, *Neottiacordata* (L.) Rich. (Kubentayev et al. 2021).

In recent years, the study of orchids of Kazakh Altai, which accounts for 22 species, has received particular attention (Danilova et al. 2020; Sumbembayev et al. 2020a, b, 2021, 2022, 2023), but orchids are still poorly studied in the northern part of Kazakhstan.

Orchid diversity in the neighbouring countries of Kazakhstan is variable. According to the latest data, there are 1,449 species in China (Zhang et al. 2015), 135 species in Russia (Efimov 2020), 26 species in Mongolia (Baasanmunkh et al. 2021), 10 species in Kyrgyzstan (Lazkov and Sultanova 2014) and nine species in Uzbekistan (Schreder 1941). Khapugin (2020), based on the synthesis of published data on the global distribution of orchids within designated conservation areas, noted the insufficient study of orchids in central and northern Asia as a whole.

Taxonomical and geographical data about orchids presented by Abdulina (1999) and earlier sources are largely outdated. Therefore, we undertook the task of preparing a new, detailed revision of this family for the flora of Kazakhstan. Taking into account that orchid family is notable for numerous rare and protected species, we have provided a detailed revision that includes lists of localities. These lists can subsequently be directly used in documents aimed at establishing the protection of the Kazakhstan flora.

The purpose of this study was to clarify the species diversity and distribution of orchids in the vast territory of the northern part of Kazakhstan, based on the revision of herbarium materials, data from literature and field observations.

## ﻿Material and method

Kazakhstan is located in the centre of Eurasia and the current ranking by area is ninth in the world with 2,724,900 km^2^. The territory of Kazakhstan is ecologically diverse, there are important zonal boundaries, including one separating the cold-temperate and temperate territories of northern Eurasia from the warm-temperate and hot-temperate territories of the Ancient Mediterranean (Abdulina 1999).

**Figure 1.**
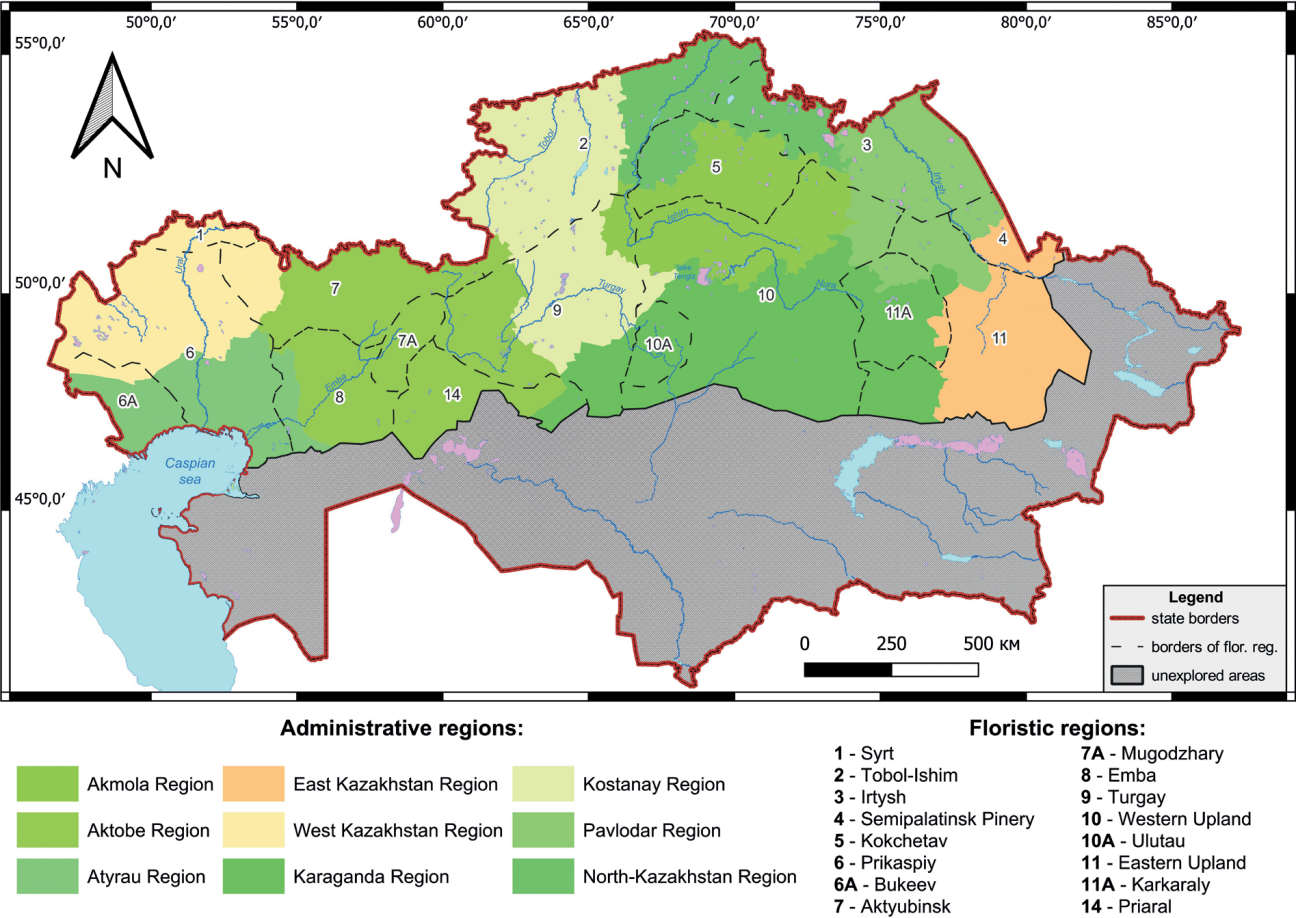
Map of floristic and administrative regions of Kazakhstan.

The presented contribution covers the major part of the country with the exception of the mountainous areas and desert areas, which are very different from the rest of the country and it is necessary to review them separately. In the article, the distribution of separate taxa is given according to both floristic and administrative principles. The studied area includes eight of 14 administrative regions (Fig. 1): Pavlodar, North Kazakhstan, Kostanay, Akmola, Aktobe, West Kazakhstan and partially Karaganda and East Kazakhstan regions). The administrative division of Kazakhstan that succeeded in 2021 is being pursued. Floristic subdivision of the territory follows Pavlov (1956). According to the latter classification, the studied area includes the following floristic regions (further abbreviated “FR”): Aktyubinsk, Bukeev, Emba, Eastern Upland, Irtysh, Karkaraly, Kokchetav, Mugodzhary, Priaral, Prikaspiy, Semipalatinsk Pinery, Syrt, Tobol-Ishim, Turgay, Ulutau and Western Upland (Fig. 1).

We have studied the following herbarium collections: LE, MW, TK, MHA, SVER, KUZ, ALTB, AA, NUR, KG, KSPI, NS, NSK, MOSP, ORIS and PPIU (acronyms according to Thiers 2022). In addition, we have studied two herbarium collections lacking acronyms: the herbarium of M. Kozybayev North Kazakhstan University, Petropavlovsk, (termed “NKU”) and the herbarium of Zhezkazgan Botanic Garden, Zhezkazgan, (termed “ZhBG”). All herbarium collections were studied either personally or after photographs.

The nomenclature of each taxon mostly follows “Plants of the World Online” (POWO 2022).

The conservation status of each species follows the Red Book of Kazakhstan (2014), which assumes three categories of rarity: I – a very rare, critically endangered species; II – a very rare species; III – a rare species with a shrinking range.

Distribution maps of individual species were prepared using ArcMap. Dubious localities (with “question-mark” in the text) are included on the maps as well.

## ﻿Results and discussion

According to our data, 25 species of orchids from 16 genera are recorded in the northern part of Kazakhstan. Eight species are listed in the Red Data Book of Kazakhstan, of which four species are classified under the II category and four species under the III category.

**Figure 2.**
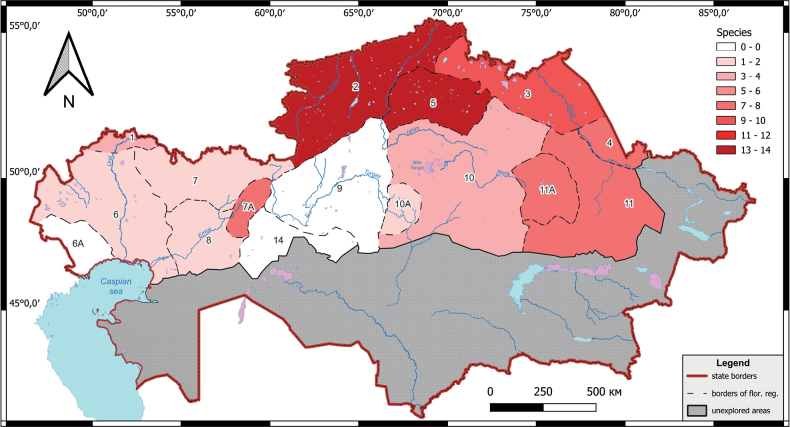
Species abundance of orchids in the floristic regions of the northern part of Kazakhstan.

The distribution of the studied species within the floristic regions is as follows: Kokchetav – 14 species, Tobol-Ishim – 13 species, Irtysh – 9 species, Mugodzhary – 8 species, Eastern Upland – 8 species, Karkaraly – 8 species, Semipalatinsk Pinery – 7 species, Aktyubinsk – 2 species, Syrt – 3 species, Western Upland – 3 species, Ulutau – 1 species, Emba – 1 species and Prikaspiy – 1 species (Fig. 2).

The larger number of species in Kokchetav, Tobol-Ishim and Irtysh floristic regions is due to the presence of the more variable spectrum of habitats for orchids, including pine, deciduous and mixed forests, river valleys, sphagnum swamps, flood meadows etc. The Mugodzhary FR, which is also relatively rich in orchids (8 species), is located in the semi-desert zone of Kazakhstan; however, the Urkach and Ber-Chugur places (“place” stands here for the Russian word “urochishche”, which is used for various vernacular toponyms and also for the names of the former settlements) are located here, which include extensive lowlands with birch-aspen forests and sphagnum swamps, a very rare type of habitat in Kazakhstan. The Urkach place is considered to be a unique remnant of fragments of northern vegetation that retreated to the north during dry interglacial times and are evidence of the former vegetation of the Mugodzhar Mountains (Rusanov 1948).

**Figure 3.**
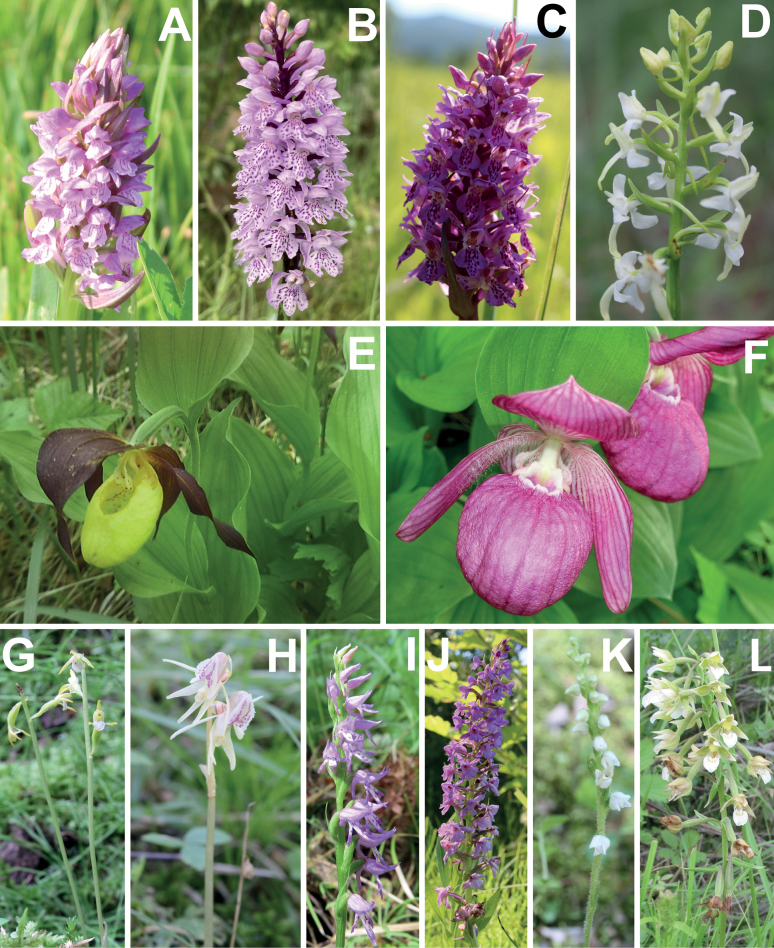
Representative taxa of Orchidaceae in the northern part of Kazakhstan **A***Dactylorhizaincarnata***B***D.fuchsii***C***D.sibirica***D***Platantherabifolia***E***Cypripediumcalceolus***F***C.macranthos***G***Corallorhizatrifida***H***Epipogiumaphyllum***I***Hemipiliacucullata***J***Gymnadeniaconopsea***K***Goodyerarepens***L***Epipactispalustris*. (Photos: **A–D, G, H, J, K** by S. Kubentayev; **E, F, I, L** by A. Kupriyanov).

Emba FR and Prikaspiy FR, where only one species of orchids (*Orchismilitaris* L.) was found, as well as Turgay FR and Priaral FR, where orchids were not found at all, represent desert and semi-desert zones of Kazakhstan, with high soil salinity. The small number of orchids in Ulutau FR (also one species, *Dactylorhizaincarnata* (L.) Soó (Figs 3A, 5B)), in our opinion, is due to the poor knowledge of this region.

The distribution of the studied species by administrative regions is the following: Akmola – 12 species, Pavlodar – 13 species, Karaganda – 10 species, Kostanay – 10 species, East Kazakhstan – 8 species, North Kazakhstan – 7 species, West Kazakhstan – 3 species and Aktobe – 8 species (Table 1).

**Table 1.** Summary of orchids distribution in the northern part of Kazakhstan.

№	Species	Number of localities	Floristic regions	Administrative regions	Category according to the Red Book of Kazakhstan
1	*Corallorhizatrifida* Châtel.	9	Kokchetav, Karkaraly	Akmola, Karaganda	–
2	*Cypripediumcalceolus* L.	21	Kokchetav, Tobol-Ishim, Irtysh, Semipalatinsk Pinery	Akmola, North Kazakhstan, Pavlodar, East Kazakhstan	III
3	*Cypripediumguttatum* Sw.	7	Tobol-Ishim, Irtysh	North Kazakhstan, Pavlodar	II
4	*Cypripediummacranthos* Sw.	5	Tobol-Ishim, Irtysh, Semipalatinsk Pinery, Kokchetav	North Kazakhstan, Pavlodar, East Kazakhstan	II
5	*Dactylorhizafuchsii* (Druce) Soó	39	Kokchetav, Tobol-Ishim, Karkaraly, Irtysh	Akmola, North Kazakhstan, Kostanay, Karaganda, Pavlodar	II
6	*Dactylorhizaincarnata* (L.) Soó	63	Tobol-Ishim, Eastern Upland, Kokchetav, Syrt, Mugodzhary, Aktyubinsk, Ulutau, Karkaraly, Western Upland, Irtysh	Kostanay, Pavlodar, North Kazakhstan, Akmola, West Kazakhstan, Aktobe, Karaganda, East Kazakhstan	–
7	*Dactylorhizamaculata* (L.) Sоó	7	Kokchetav, Karkaraly, Mugodzhary	Akmola, Karaganda, Aktobe	–
8	*Dactylorhizasalina* (Turcz. ex Lindl.) Soó	6	Eastern Upland, Western Upland, Tobol-Ishim	Karaganda, Kostanay	–
9	*Dactylorhizasibirica* Efimov	2	Eastern Upland	Pavlodar, East Kazakhstan	–
10	*Dactylorhizaumbrosa* (Kar. & Kir.) Nevski	7	Kokchetav, Tobol-Ishim, Mugodzhary, Western Upland, Eastern Upland	Akmola, Kostanay, Aktobe, Karaganda, East Kazakhstan	–
11	*Epipactisatrorubens* (Hoffm.) Besser	2	Tobol-Ishim	Kostanay	–
12	*Epipactishelleborine* (L.) Crantz	1	Mugodzhary	Aktobe	–
13	*Epipactispalustris* (L.) Crantz	17	Aktyubinskiy, Mugodzhary, Syrt, Tobol-Ishim, Kokchetav, Semipalatinsk Pinery, Karkaraly, Irtysh	Aktobe, West Kazakhstan, Kostanay, Akmola, Pavlodar, East Kazakhstan, Karaganda	III
14	*Epipogiumaphyllum* Sw.	1	Karkaraly	Karaganda	II
15	*Goodyerarepens* (L.) R.Br.	12	Kokchetav	Akmola	–
16	*Gymnadeniaconopsea* (L.) R.Br.	24	Kokchetav, Tobol-Ishim, Irtysh, Semipalatinsk Pinery	Akmola, Kostanay, North Kazakhstan, Pavlodar, East Kazakhstan	–
17	*Hammarbyapaludosa* (L.) Kuntze	1	Mugodzhary	Aktobe	–
18	*Hemipiliacucullata* (L.) Y.Tang, H.Peng & T.Yukawa	4	Kokchetav, Eastern Upland	Akmola, Pavlodar	–
19	*Herminiummonorchis* (L.) R.Br.	1	Semipalatinsk Pinery, Irtysh	East Kazakhstan, Pavlodar	–
20	*Liparisloeselii* (L.) Rich.	3	Kokchetav, Mugodzhary, Semipalatinsk Pinery	Akmola, Aktobe, East Kazakhstan	–
21	*Malaxismonophyllos* (L.) Sw.	8	Tobol-Ishim, Eastern Upland, Karkaraly	Kostanay, Pavlodar, Karaganda	–
22	*Neottiacamtschatea* (L.) Rchb.f.	5	Karkaraly, Eastern Upland	Karaganda, Pavlodar	–
23	*Orchismilitaris* L.	11	Prikaspiy, Mugodzhary, Syrt, Semipalatinsk Pinery, Eastern Upland, Emba	West Kazakhstan, Aktobe, East Kazakhstan	III
24	*Platantherabifolia* (L.) Rich.	20	Tobol-Ishim, Kokchetav	Kostanay, North Kazakhstan	III
25	*Spiranthesaustralis* (R.Br.) Lindl	4	Tobol-Ishim, Kokchetav, Irtysh	Kostanay, Akmola, Pavlodar	–

**Figure 4.**
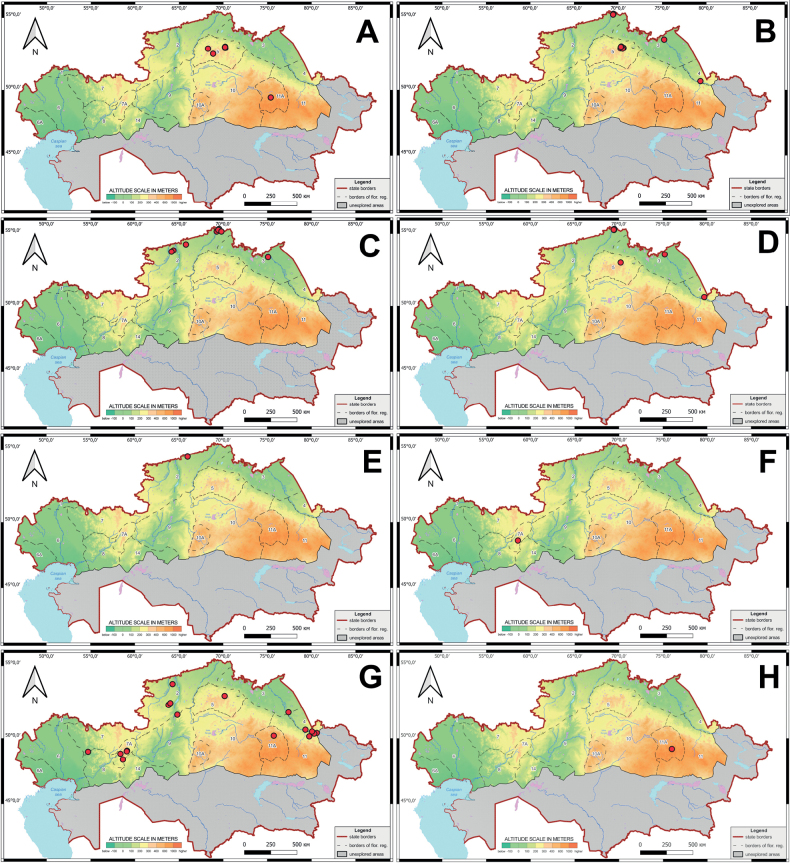
Schematic map of the localities of orchids in the northern part of Kazakhstan **A***Corallorhizatrifida***B***Cypripediumcalceolus***C***C.guttatum***D***C.macranthos****E****Epipactisatrorubens***F***E.helleborine***G***E.palustris***H***Epipogiumaphyllum*.

**Figure 5.**
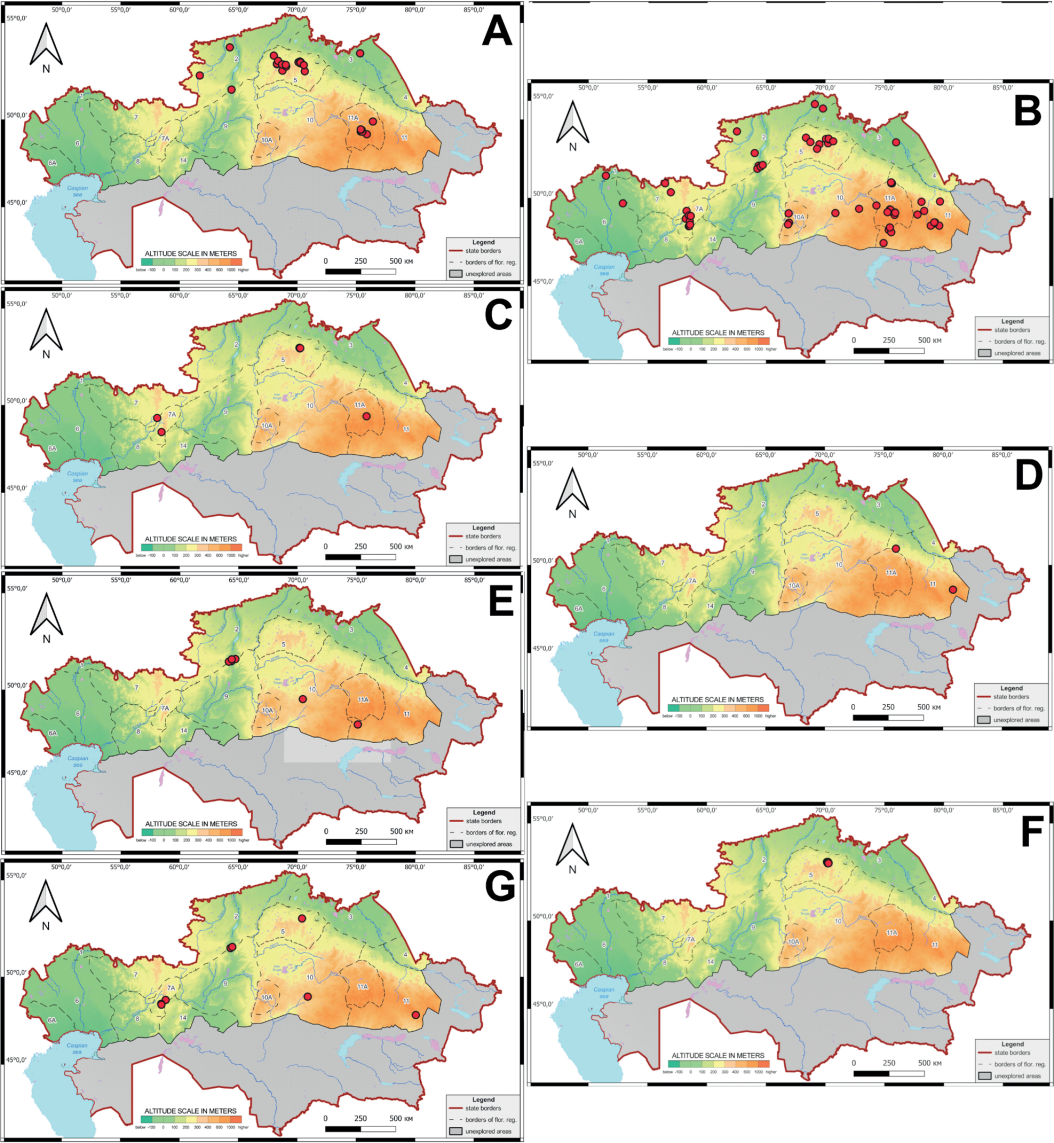
Schematic map of the localities of orchids in the northern part of Kazakhstan **A***Dactylorhizafuchsii***B***D.incarnata***C***D.maculata***D***D.sibirica***E***D.salina***F***G.repens***G***D.umbrosa*.

**Figure 6.**
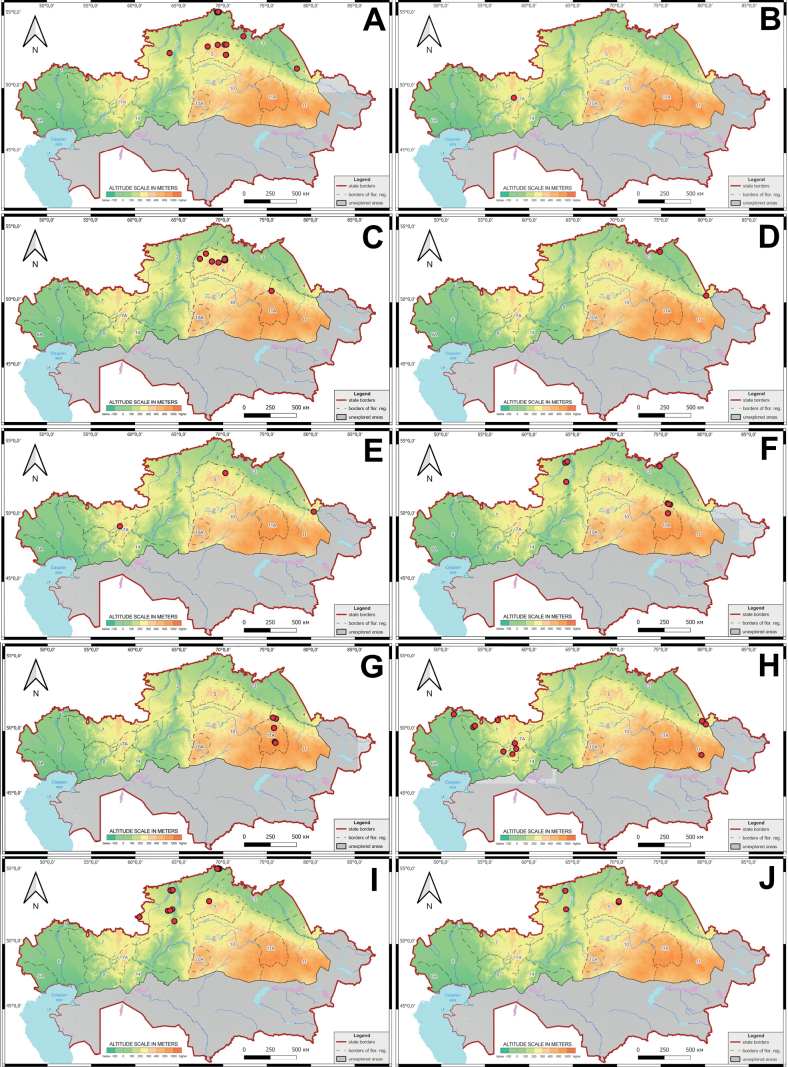
Schematic map of the localities of orchids in the northern part of Kazakhstan **A***Gymnadeniaconopsea***B***Hammarbyapaludosa***C***Hemipiliacucullata***D***Herminiummonorchis***E***Liparisloeselii***F***Malaxismonophyllos***G***Neottiacamtschatea***H***Orchismilitaris***I***Platantherabifolia***J***Spiranthesaustralis*.

Currently, eight species of orchids growing in the northern part of Kazakhstan are included in the Red Book of Kazakhstan (2014): *Cypripediumcalceolus* L. (Figs 3E, 4B), *C.guttatum* Sw., *C.macranthos* Sw. (Figs 3F, 4D), *Dactylorhizafuchsii* (Druce) Soó (Figs 3B, 5A), *Epipactispalustris* (L.) Crantz (Figs 3L, 4G), *Epipogiumaphyllum* Sw. (Figs 3H, 4H), *Orchismilitaris* and *Platantherabifolia* (L.) Rich (Figs 3D, 6I). We recommend to additionally include in the next edition of the Red Book of Kazakhstan nine species, viz. *Corallorhizatrifida* Chatel. (Figs 3G, 4A), *Epipactisatrorubens*, *Gymnadeniaconopsea* (L.) R.Br. (Figs 3J, 6A), *Hammarbyapaludosa*, *Herminiummonorchis* (L.) R.Br., *Liparisloeselii* (L.) Rich., *Malaxismonophyllos* (L.) Sw., *Neottiacamtschatea* (L.) Rchb. f. and *Spiranthesaustralis* (R.Br.) Lindl. Thus, 17 species of orchids growing in the studied region should be included in the next edition of the Red Book of Kazakhstan.

Based on our research, we discovered a single herbarium specimen from the Mugodzhary FR. In our assessment, it appears to be *Dactylorhizaochroleuca* (Wüstnei ex Boll) Holub. However, this finding requires confirmation, as there is a possibility of confusion with hypochromic variants of *Dactylorhizaincarnata*. *Dactylorhizasibirica* Efimov (Figs 3C, 5D) is reported for the first time for the northern part of Kazakhstan. Many taxa are reported for the first time for particular floristic and administrative regions of the country.

The most widespread species in the studied region are *Dactylorhizaincarnata* (63 localities in 10 FR), *Epipactispalustris* (17 localities in 8 FR), *Dactylorhizaumbrosa* (Kar. & Kir.) Nevski (7 localities in 5 FR). The rarest species (one location only) are *Epipactisatrorubens*, *Epipactishelleborine* (L.) Crantz, *Epipogiumaphyllum*, *Hammarbyapaludosa* and *Herminiummonorchis* (Table 1).

*Dactylorhizamajalis* (Rchb.) P.F. Hunt & Summerh and *D.russowii* (Klinge) Holub, reported earlier for the studied region (Pavlov 1956; Aipeisova 2012, 2013; Kupriyanov 2020), are excluded from the flora of Kazakhstan as erroneous determinations. More recently, Sumbembayev et al. (2023) reported Dactylorhiza×kerneri (Soó) Soó (= *D.fuchsii* × *D.incarnata*) for the flora of Kazakhstan, based on herbarium collections stored in LE. We believe that those specimens can be rather safely determined as *Dactylorhizasibirica*, a hybridogenous species originating according to the same hybrid formula.

### ﻿ Corallorhiza trifida

Taxon classificationPlantaeAsparagalesOrchidaceae

Châtel.

50691C44-7BF3-5F06-9F99-210F72106BD2

#### Distribution in adjacent reg.

Russia (European Russia, Ural, Siberia), Kazakhstan (Altai, Western Tien Shan).

#### Specimens examined and literature records.

Kokchetav: **Akmola Region**: Sandyktau District: Maraldy, near the village of Sandyktau, 5 Jul 1913, *Semenov s.n..* (TK!); Burabay District: “Burabay” State National Nature Park: east shore of Shchuchye Lake, 17 Jun 1972, *Gorchakovskiy s.n..* (SVER 695750!); upper reaches of Imanaevskiy Spring, 2 Aug 1895, *Gordiagin 594* (LE!); near Burabay, 12 Jun 1913, *Drobov 430* (LE!); near Karas’e Lake, 27 Jun 1901, *Gordiagin 514* (LE!); same loc., 20 Jun 2012, *Khrustaleva and Artemova s.n..* (KUZ 02684!); near of Svetloe Lake, 15 Jul 1960, *Denisova 1577* (MW 0816955!); Aiyrtau District: Kokshetau State National Nature Park, Imantau Mountains, “Buyan-Schel” place, 31 May 1973, *Gorchakovskiy s.n..* (SVER 695749!). Karkaraly: **Karaganda Region**: Karkaraly District: Karkaraly Mountains, Alexandrov Klyuch cordon, 31 May 2007, *Kupriyanov et al. s.n..* (KUZ 11464!).

#### Habitat and ecology.

Forest swamps, stream valleys, lakesides, sphagnum swamps and wet birch forests.

#### Phenology.

Flowering in May–Jun; fruiting in Jul–Aug.

#### Conservation status.

Not protected. We recommend to include this species in the next edition of the Red Book of Kazakhstan.

#### Notes.

*Corallorhizatrifida* is reported for the Kokshetau State National Nature Park for the first time.

### ﻿ Cypripedium calceolus

Taxon classificationPlantaeAsparagalesOrchidaceae

L.

3C107815-3A16-5E80-8BE8-93BAC661D5CC

#### Distribution in adjacent reg.

Russia (European Russia, Ural, Siberia), Kazakhstan (Altai).

#### Specimens examined and literature records.

Kokchetav: **Akmola Region**: Burabay District: “Burabay” State National Nature Park: near Shchuchinsk, Barmashinskoe forestry, 12 Jul 2019, *Kubentaev s.n..* (NUR!); same loc., 15 Jun 1965, *Oleneeva and Antoshenko s.n..* (SVER 695751!); same loc., 18 Jun 2012, *Artemova s.n..* (KUZ 02637!); same loc., 8 Jun 2011, *Kupriyanov and Hrustaleva s.n..* (KUZ 01096!); same loc., 2.5 km northeast of Shchuchinsk, 12 Jun 2011, *Hrustaleva s.n..* (KUZ 00884!); Zolotoborskoe forestry, 23 Jun 2016, *Hrustaleva and Artemova s.n..* (KUZ 02798!); same loc., 3 km south of the Barmashino, 13 Jun 1972, *Gorchakovskiy s.n..* (SVER 695752!); near Barmashino Lake, 26 Jun 1890, *Gordyagin 503* (LE!); the northern shore of Kotyrkol Lake, 19 Jun 1902, *Ignatov and Petrovskiy 209* (LE!); same loc., 31 May 1902, *Ignatov and Petrovsky 209* (LE!); located 3–3.5 km east of Katarkol (Kupriyanov 2020); located 2.5 km northeast of Burabay, on the shore of Borovoe Lake (Kupriyanov 2020). Tobol-Ishim: **North Kazakhstan Region**: Kyzylzhar District: on the right shore of Ishim River, 75 km north of Petropavlovsk, 5 km north of Krasnoyarka, 16 Jun 1968, *Kolodchenko s.n..* (АА!, LE!); on the right shore of Ishim River, near Krasnoyarka, Jun 1968, *Syzganov et al. s.n..* (NKU!); same loc., Jun 1968, *Terekhina et al.* s.n.. (NKU!); same loc., Jun 1968, *Freze s.n..* (NKU!). Irtysh: **Pavlodar Region**: Zhelezinskiy District: on the right shore of Irtysh River (Kusnetsov and Pavlov 1958; Kazakh SSR Red Data Book 1981). Semipalatinsk Pinery: **East Kazakhstan Region**: Beskaragay District: on the right shore of Irtysh River (Kusnetsov and Pavlov 1958; Kazakh SSR Red Data Book 1981; Red Book of Kazakhstan 2014).

#### Habitat and ecology.

Birch and birch-pine forests, forest stream valleys, forest swamps and forest lake shores.

#### Phenology.

Flowering in Jun; fruiting in Jul–Aug.

#### Conservation status.

It is included in the Red Book of Kazakhstan (category III). It is a rare and endangered species protected in the “Burabay” State National Nature Park, “Sogrov” State Nature Reserve and “Floodplain of the Irtysh River” State Nature Reserve.

#### Notes.

Some populations of *Cypripediumcalceolus* are located near Shchuchinsk and the village of Burabay, in areas with high recreational activity. These populations require special attention and protection due to the low number of plants in the populations, which can be attributed to the significant anthropogenic impact in these areas (Sultangazina et al. 2014; Kupriyanov 2020).

### ﻿ Cypripedium guttatum

Taxon classificationPlantaeAsparagalesOrchidaceae

Sw.

941D192A-C356-51A8-A86B-8041E8DA2A06

#### Distribution in adjacent reg.

Russia (European Russia, Ural, Siberia), Kazakhstan (Altai).

#### Specimens examined and literature records.

Tobol-Ishim: **North Kazakhstan Region**: Kyzylzhar District: on the right shore of Ishim River, approximately 75 km north of Petropavlovsk and 5 km north of Krasnoyarka, 16 Jun 1968, *Syzganov and Sadvokasova s.n..* (LE); the left shore of Ishim River, near Krasnoyarka, 17 Jun 1968, *Sidarkina and Galieva s.n.* (NKU!); near Vagulino, 12 Jun 1982, *Rain and Martyasheva s.n..* (NKU!); near Tashkentka, 25 Jun 1982, *Vafina et al. s.n.* (NKU!). ? **Kostanay Region**: ?Uzynkol District (Pugachev 1994), ?Mendykara District (Pugachev 1994). Irtysh: **Pavlodar Region** [without detailed locality] (Kusnetsov and Pavlov 1958).

#### Habitat and ecology.

Wet birch forests.

#### Phenology.

Flowering in Jun; fruiting in Jul–Aug.

#### Conservation status.

This rare species is included in the Red Book of Kazakhstan (category II) and is protected within the territory of two State Nature Reserves: “Sogrov” and “Floodplain of the Irtysh River”.

#### Notes.

The report of *Cypripediumguttatum* for the Kostanay Region is doubtful since we have not found herbarium collections from these areas, including the herbarium of Kostanay Pedagogical University (KSPI), where the Pugachev collections are stored.

### ﻿ Cypripedium macranthos

Taxon classificationPlantaeAsparagalesOrchidaceae

Sw.

40291F74-E7A4-5866-9CA2-C5A7CE538186

#### Distribution in adjacent reg.

Russia (European Russia, Ural, Siberia), Kazakhstan (Altai).

#### Specimens examined and literature records.

Tobol-Ishim: **North Kazakhstan Region**: Kyzylzhar District: on the right shore of Ishim River, 5 km north of Krasnoyarka, 16 Jun 1968, *Kolodchenko s.n..* (АА!, LE!); on right shore of Ishim River, near Krasnoyarka, Jun 1968, *Shakarova et al. s.n..* (NKU!); same loc., 27 Jun 1987, *Samoylova et al. s.n..* (NKU!). ?Kokchetav: ?**Akmola Region** [without detailed locality] (Semenov 1928; Kusnetsov and Pavlov 1958; Gorchakovskiy 1987). Irtysh: **Pavlodar Region** [without detailed locality] (Kusnetsov and Pavlov 1958). Semipalatinsk Pinery: **East Kazakhstan Region**: Beskaragay District: near Kara-Murza, 16 Jun 1956, *Olovitikova s.n..* (LE!).

#### Habitat and ecology.

Birch forests and valleys of forest streams.

#### Phenology.

Flowering in Jun; fruiting in Jul–Aug.

#### Conservation status.

This very rare species is included in the Red Book of Kazakhstan (category II). It is protected within the “Sogrov” and “Floodplain of the Irtysh River” State Nature Reserves.

#### Notes.

According to recent reports (Sultangazina et al. 2014; Kupriyanov 2020) and our field studies, there is currently no confirmation of the presence of *Cypripediummacranthos* within the territory of Kokchetav FR.

One specimen of *C.×ventricosum* (*C.calceolus × C.macranthos*) hybrid was found: “Tobol-Ishim: North Kazakhstan Region: Kyzylzhar District: on the right shore of the Ishim River, near Krasnoyarka, 17 Jun 1968, *Tsykareva s.n..* (AA!)”. This species occurs in areas where parent species co-occur, forming transitional populations with intermediate morphology (Averyanov 1999; Knyazev et al. 2000; Filippov and Andronova 2011; Andronova et al. 2017). This hybrid is reported for the first time in the studied region; Previously it was only reported in the Katon-Karagai District of the East Kazakhstan region in Kazakhstan (Kotuhov et al. 2009, 2018).

### ﻿ Dactylorhiza fuchsii

Taxon classificationPlantaeAsparagalesOrchidaceae

(Druce) Soó

A94DE8F2-832B-5B2D-A277-E3823F7C3CF6

(=Dactylorhizahebridensis (Wilmott) Aver., ≡Dactylorhizafuchsiisubsp.hebridensis (Wilmott) Soó).

#### Distribution in adjacent reg.

Russia (European Russia, Ural, Siberia), Kazakhstan (Altai).

#### Specimens examined and literature records.

Kokchetav: **Akmola Region**: Burabay District: Near Karas’e Lake, 4 Jul 1937, *Shishkina s.n.* (AA!); same loc., Zolotoborskiy forestry, 21 Jun 1972, *Gorchakovskiy s.n..* (SVER 695767!); Borovskoy forest area, upper reaches of Imanayevsky Creek, 27 Jun 1974, *Gorchakovskiy s.n..* (SVER 695769!); same loc., 16 Jun 1972 *Gorchakovskiy s.n..* (SVER 695769!); Barmashinskiy experimental forestry, 1 Aug 1965, *Tyulebergeneva s.n*. (SVER 695763!); Zolotoborsky forestry, eastern shore of Shchuchye Lake, 3 km north of the Medvezhiy cordon, 17 Jun 1972, *Gorchakovskiy s.n..* (SVER 695756!); Burabay forest area, near Akylbay cordon, 10 Jun 1913, *Drobova 308* (LE!); Burabay forest area, near Dorofeyevka, 10 Jun 1913, *Drobova 309* (LE!); “Burabay” State National Nature Park: Borovskoe forestry, 16 Jun 2019, *Kubentayev and Alibekov s.n..* (NUR!); Barmashinskoe forestry, 7 Aug 2020, *Kubentayev et al. s.n..* (NUR!); Temnoborskoe forestry, near Zhukey Lake, north-west coast, 11 Jun 2019, *Kubentayev et al. s.n..* (NUR!); near Burabay, the headwaters of Imanayevsky Creek, 7 Jun 2011, *Kupriyanov s.n..* (KUZ 00816!); near Makinka, 11 Jun 2011, *Kupriyanov s.n..* (KUZ 01111!); Zerendi District: “Kokshetau” State National Nature Park: Zerendi forestry, near Zerenda, 28 May 2020, *Kubentayev and Alibekov s.n..* (NUR!); Oramndybulakskoe forestry, near Karsak, 27 Jun 2020, *Kubentayev and Alibekov s.n..* (NUR!); Zerendi forestry, near Krasniy cordon, 26 Jun 2019, *Kubentayev et al. s.n..* (NUR!); Sandyktauskiy District: Sandyktau forestry, near “Chernichniy log”, 16 Jun 1957, *Gribanov s.n..* (AA!). **North Kazakhstan Region**: Aiyrtau District: Imantau Mount, 7 Aug 1965, *Oleneva and Antoshenko s.n..* (695765 SEVR!); same loc., the valley of the stream flowing down from Imantau Mount, 30 May 1973, *Gorchakovskiy s.n..* (SEVR 695760!); Imantau Mount, Bayan Gorge, 28 Aug 1981, *Gorchakovskiy s.n..* (SEVR 627680!); “Kokshetau” State National Nature Park, near Syrymbet, 10 Aug 2020, *Kubentayev et al. s.n..* (NUR!); near Lobanovo, 27 Jul 2019, *Kubentayev et al. s.n..* (NUR!). Tobol-Ishim: **Kostanay Region**: Mendykara District: botanical nature monument “Planting of birch and pine forests near Borovskoye Lake”, Jun 2009, *Perezhogin* (personal observation); Denisovskiy District: Ordzhonikidzevskoye forestry, near Denisovka (Pugachev 1994); Naurzum District: in the “Naurzum” State Nature Reserve, s.d., *KSPI students* (SVER 507474!). Karkaraly: **Karaganda Region**: Karkaraly Mountains, 27 Jun 1843, *Schrenk s.n..* (AA!); same loc., 21 Jun 1890, *Korzhinsky s.n..* (LE!); same loc., 1927, *Melville s.n..* (АА!), near Karkaraly, 3 Jul 1937, *Dmitrieva s.n..* (AA!); same loc., same loc., 17 Jul 1987, *Kupriyanov s.n..* (KG!); same loc., 18 Jun 2001, *Ishmuratova s.n* (KG!); the shore of Pashennoe Lake, 20 Jun 1914, *s. collector 2071* (LE); 70 km southeast of Karkaraly, Kent Mountains, 19 Jul 1968, *Rachkova 784* (LE!); Karkaraly Mountains, Alexandrov Klyuch, 8 Jul 1993, *Mikhailov s.n..* (KG!); Karkaraly Mountains, Karkaralinka River valley, 5 Aug 1989, *Denisova 692* (MW 0816794!). Irtysh: **Pavlodar Region** [without detailed locality] (Kusnetsov and Pavlov 1958).

#### Habitat and ecology.

Moist pine and birch forests, along the shores of forest streams and lakes, forest swamps.

#### Phenology.

Flowering in Jun–Jul; fruiting in Aug–Sep.

#### Conservation status.

It is included in the Red Book of Kazakhstan (category II). It is protected on the territories of the “Naurzum” State Nature Reserve; “Kokshetau” State National Nature Park, “Burabay” State National Nature Park, Karkaraly State National Nature Park; Botanical nature monument “Planting of birch and pine forests near Borovskoye Lake”.

#### Notes.

During the revision of KUZ herbarium materials, we identified two specimens of *D.fuchsii*, that were previously incorrectly determined as *Dactylorhizarussowii*. Based on these incorrectly misidentified herbarium specimens, *D.russowii* was previously reported for the Kokchetav FR (near Burabay, the headwaters of Imanayevsky Creek; near Makinka) and in the overall flora of Kazakhstan (Kupriyanov 2020). As a result, there is currently no reliable information regarding the presence of *D.russowii* in Kazakhstan.

### ﻿ Dactylorhiza incarnata

Taxon classificationPlantaeAsparagalesOrchidaceae

(L.) Soó

391C9374-A15B-5AC4-B581-63BD8AED381D

#### Distribution in adjacent reg.

Russia (European Russia, Ural, Siberia), Kazakhstan (Altai, Western Tien Shan, Betpak Dala, Balkhash-Alakol Basin, Turanskaya lowlands).

#### Specimens examined and literature records.

Tobol-Ishim: **Kostanay Region**: Auliekol District: Aman-Karagay pine forest, 1930, *Dmitrieva s.n..* (AA!); Naurzum District: Naurzum-Karagai Mountains, 20 May 1909, *Savich and Kucherovskaya 647* (TK!); Naurzum Reserve, the shore of Small Aksuat Lake, 2 Jul 1949, *Ivleva and Kleshchina s.n.* (TK!); 20 km east of Aksuat, in the upper reaches of Akbulak Stream, 12 Jun 1936, *Voronov 157* (MW 0816813!); Moss swamp amongst springs on the slope of the Ulkendamdy River Valley, 23 Jun 1909, *Kucherovskaya 1125* (LE!); Fedorov District: Between the Traktov and Nazaryev, on the edge of the lake basin (Uballa Lake), 7 Jul 1913, *Korotkiy and Lebedeva s.n..* (LE!). Eastern Upland: **Pavlodar Region**: Bayanaul District: “Bayanaul” State National Nature Park, Bayanaul Mountains, 27 May 2007, *Kupriyanov et al. s.n..* (KUZ 11467!). **North Kazakhstan Region**: Gabit Musrepov District: near the “Ardager” recreation centre, 27 Jun 2019, *Kubentayev s.n..* (NUR!); the City of Petropavlovsk, near Pestroye Lake, 10 Jun 1962, *Troinikova 1336* (MW 0816799!); Akkain District: west of the Borki Village, 29 Jun 1979, *Kolodchenko s.n..* (NKU!). **Karaganda Region**: Aktogay District: the southern tip of the Kyzyl-Rai mountain system, the floodplain of the drying Karatal River, 2 Jul 1969, *Mishchenkova 360* (LE!); same loc., 14 Jul 1974, *Denisova 57* (LE!); same loc., in the Zhenishke River Valley, 27 Jul 1959, *Denisova 224* (LE!); Bektau ata Mountains, *Mikhailov and Alibekov s.n..* (KG!). **East Kazakhstan Region**: Abay District: the Sherubai Valley, Nura River on the shore of the Topar Reservoir, Jun 2006, *Kupriyanov s.n..* (KG!); Degelen Mountains, along the shore of the Uzynbulak Stream, 29 May 1910, *Kucherovskaya 309* (LE!); near the Ak-Jaman Mountains (Zhamantas), 4 Jun 1914, *Shipchinsky 572* (LE!); same loc., 21 Jun 1984, *Grubov et al., 603* (LE!); Chingiz Mountains, Kopa River Valley, 30 May 1914, *Kosinskiy 558* (LE!); the valley of the Chagan River located 1.5 km above the mouth of the Saryzhal River, 14 Jul 1984, *Korobkov 603* (LE!); Chingiztau Mountains upper Bakanas, the sources of the Kyzyluzen on the Barshatas-Abai road, 11 Jun 1984, *Grubov 316* (LE!); Chingiztau Mountains, Bakanas Valley, near Ramadan Village, 9 Jun 1984, *Korobkov 221* (LE!); valley of Namaz River, 28 May 1914, *Kossinsky 485* (LE!); Chingiz Mountains, Munar River Valley, 27 May 1914, *Shipchinsky 345* (LE!); Abraly District: valley between the Zhaksyabraly and Zhamanabraly Mountains, 4 Jun 1910, *Kucherovskaya 668a* (LE!); Semipalatinsk District: the western part of the mountain (Kokon) Kokentau, 15 Jun 1928, *Blumenthal and Zapryagaev 475* (LE!). Kokchetav: **Akmola Region**: Zerendi District: near Kostomarovka, 4 Jun 1986, *Mikhailov s.n..* (KG!); Archaly is 5 km from Lesogorskoe Village, 24 Jun 1929, *Grigoriev s.n..* (AA!); “Kokshetau” State National Nature Park, Ormandy Bulak forestry, near Ermakovka, 28 May 2020, *Kubentayev s.n..* (NUR!); Burabay District: near Dorofeevka (Akylbay), 3 Jun 1918, *Drobov 305* (LE); to the SE from Shchuchya station, 28 Jun 1997, *Gordyagin 20* (LE!); Burabay, Rashit - cordon, along the shore of Arykpay River, 9 Jun 2011, *Khrustaleva s.n..* (KUZ 00969!); “Burabay” State National Nature Park, 101 block of Borovskoy forestry, 22 Jun 2012, *Khrustaleva and Artemova 02776* (KUZ!); Zolotoborsky forestry, near Tas-Shalkar Lake, 23 Jun 2012, *Khrustaleva and Artemova s.n..* (KUZ 02810!). SYRT: **West Kazakhstan Region**: Terekti District: near Podstepny to the southwest of Uralsk, 1895, *Kulyasov 51* (MW 296063!); near Temirbek, 13 Jun 1908, *Borodin et al. s.n..* (LE!). Mugodzhary: **Aktobe Region**: Mugalzhar District: the southern tip of the Mugodzhar Mountains, west of Algabas railway station, 21-22 Jun 1987, *Skvortsov s.n..* (MHA!); horse farm Emba, 1934, *Buyanov s.n..* (MHA!); Near the north-west of the Mugodzhar Mountains, in a meadow near the sands of Urkach, *Dubinskaya 84* (LE!); Bol’shoy Boktybay Mountains, Berchogur place, 8 Jul 1927, *Rusanov 372* (LE!); The upper reaches of the Chili River (Or) near of the place Dzhaksy-Urkach, 11 Jul 1927, *Krasheninnikov 847* (LE!); To the NW from Berchogur Station on the way to Mugojar, 25 Jun 1925, *Krasheninnikov 576* (LE!); at the foot of Dau-Tau Mountain, 14 Jun 1936, *Khomutova and Daeva s.n..* (MW 0816798!); near the Ayryuk Mountain, 3 Jul 1927, *Krasheninnikov 757* (LE!). Aktyubinsk: **Aktobe Region**: Martuk District: 7 km from Martuk, 17 Jun 1993, *Panina s.n.* (PPIU); Khobdinsky District: near Bestau Mount, 23 Jul 1934, Semsel 159 (MW 0816811!). Ulutau: **Karaganda Region**: Ulytau District: near Ulytau Mountains, 1842, *Schrenk s.n..* (LE!); same loc., 2 Jun 2016, *Nashenova and Ivanov s.n..* (ZhBG!); Arganaty Mountains, 27 Jun 2017, *Kupriyanov and Khrustaleva s.n..* (KUZ 08448!); same loc., floodplain of the River Bazoy, 27 Jun 2017, *Kupriyanov and Khrustaleva s.n..* (KUZ 08449!). Karkaraly: **Karaganda Region**: Karkaraly District: on the salty meadows near the Big Lake, 23 Jul 1890, *Korzhinskiy s.n..* (LE!); Karkaraly Mountains 14 km south of Karkaralinsk, 14 Jun 1959, *Denisova s.n..* (LE!); Karkaraly Mountains on the road between Karkaraly and Zharly, 22 Jun 1991, *Pimenov and Klyukov 29* (MW 0816812!); near Karkaralinsk, near Zhyrym River, 12 May 1914, *Kucherovskaya 1697* (LE!); near Kent Village, Kent Mountains, 2 Jun 2007, *Kupriyanov et al. s.n..* (KUZ 11468!); same loc., 16 Jun 2001, *Ishmuratova s.n.* (KG!). Western Upland: **Karaganda Region**: Abay District: the shore of Talda River, near Amanzholov Farm, 20 Jun 1993, *Mikhailov s.n..* (KG!); Akbastau River Valley, 3 May 2015, *Alibekov s.n..* (KG!); the valley of the Sherubai, Nura River at the shore of the Topar Reservoir, Jun 2006, *Kupriyanov s.n..* (KG!); Shetskiy District: Shopa River Valley, foothills Kusmuryn, 25 Jun 1994, *Mikhailov s.n..* (KG!).

#### Habitat and ecology.

Wet meadows, flooded saline meadows, valleys of rivers, streams, lakeshores.

#### Phenology.

Flowering in Jun–Jul; fruiting in Aug–Sep.

#### Conservation status.

Not protected.

#### Notes.

*Dactylorhizaincarnata* is the most common orchid species in the studied region, found in almost all areas. However, for Turgay FR, it was recently reported by mistake. The corresponding localities (Sumbembayev et al. 2023) actually refer to the Tobol-Ishim FR. There are currently no known orchid species that occur in Turgay FR. Existing reports of *D.traunsteineri* (Saut. ex Rchb.) Soó for Kazakhstan are most likely based on misidentified *D.incarnata.* Therefore, the report of *D.traunsteineri* for Urkach Plateaeu in Mugodzhary (Kusnetsov and Pavlov 1958) is possibly based on a herbarium specimen in LE with incomplete label (Alexandri Lehmann Reliquiae botanicae, *Orchisangustifolia*, [det.] Al. Bunge), which was probably collected near the end of May 1842 (Bunge 1847).

During the revision of MHA herbarium materials, we found a herbarium specimen from the Mugodzhary FR (Aktobe Region: Mugalzhar District: the southern tip of the Mugodzhar Mountains, west of Algabas railway station, 21-22 Jun 1987, Skvortsov s.n.. (MHA!)). In our opinion, this specimen corresponds to *Dactylorhizaochroleuca*. The identification is based on information provided on the herbarium label (“pale, pink-fawn flowers”). However, it is possible that these plants belong to hypochromic variants of *D.incarnata*. Exact determination is possible only through allozyme analysis (Filippov et al. 2017) or DNA analysis.

### ﻿ Dactylorhiza maculata

Taxon classificationPlantaeAsparagalesOrchidaceae

(L.) Sоó

4B386E95-6604-5B93-9DBB-498D5375892B

#### Distribution in adjacent reg.

Russia (European Russia, Ural, Siberia), Kazakhstan (?Altai).

#### Specimens examined and literature records.

Kokchetav: **Akmola Region**: Burabay District: “Burabay” State National Nature Park: the shore of Svetloye Lake, 8 Jun 1960, *Denisova 1326* (MW 0816814!); Barmashinsky forestry, 7 Jun 2019, *Kubentayev s.n..* (NUR!); same loc., the planning quarter 134, 18 Jun 2012, *Artemova* (KUZ 02650!); the swampy shore of Karas’e Lake, *Khrustaleva and Artemova s.n..* (KUZ 02696!). Karkaraly: **Karaganda Region**: Karkaraly District: Karkaraly Mountains, 12 Aug 2006, *Kupriyanov and Manakov s.n..* (KUZ 11465!). Mugodzhary: **Aktobe Region**: Mugalzhar District: Mugodzhary Mts, “Urkach” place (Aipeisova 2013); Shalkar District: near Ber-Chugur railway station (Aipeisova 2013).

#### Habitat and ecology.

Sphagnum swamps.

#### Phenology.

Flowering in Jun–Jul; fruiting in Aug–Sep.

#### Conservation status.

Not protected.

#### Notes.

*Dactylorhizamaculata* is often hardly distinguishable from *D.fuchsii*. When they co-occur, they form populations that include plants with intermediate morphology, indicating possible hybridisation. We consider that *D.maculata* is generally a European species, with only isolated occurrences in Asia, particularly in the western part of Siberia and in Kazakhstan. Determining the exact eastern distribution limit of this species is challenging due to its similarity with *D.fuchsii* in this region, where their ranges overlap.

### ﻿ Dactylorhiza salina

Taxon classificationPlantaeAsparagalesOrchidaceae

(Turcz. ex Lindl.) Soó

437D2305-3B48-52A1-A053-27885585CD24

#### Distribution in adjacent reg.

Russia (Siberia), Kazakhstan (Altai, Western Tien Shan, Turanskaya lowlands).

#### Specimens examined and literature records.

Eastern Upland: **Karaganda Region**: Aktogay District: near Aktogay, 24 Jun 1917, *Harin s.n..* (AA!). Western Upland: **Karaganda Region** (without detailed locality) (Kupriyanov 2020). Tobol-Ishim: **Kostanay Region**: Naurzum District: “Naurzum” State Nature Reserve, near Biragach, 8 Jun 1984, *Zaugol’nova s.n..* (MOSP!); near Naurzum-Karagai, 20 May 1909, *Savich and Kucherovskaya s.n..* (LE!); near Karamenda, the shore of Sarymoyin Lake, 1 Jul 1911, *Borodin s.n..* (LE!); Egin-Bulak spring, north of Naurzum Forest, 22 Jun 1934, *Pavlov 1396* (MW 0816830!); 5 km south of Aksuat Village, 20 Jun 1945, *Voronov s.n..* (MW 0816882!).

#### Habitat and ecology.

Lowlands amongst birch trees, damp saline meadows and floodplains of rivers.

#### Phenology.

Flowering in Jun–Jul; fruiting in Aug–Sep.

#### Conservation status.

Not protected.

#### Notes.

*Dactylorhizasalina* is reported here for the first time for the Tobol-Ishim FR and Kostanay Region. *D.salina* was recently erroneously reported for the Turgay FR (Sumbembayev et al. 2023); in fact, the corresponding localities refer to the Tobol-Ishim FR. There are currently no orchid species known to occur in the Turgay FR. *D.salina* is hardly distinguishable from *D.umbrosa*.

### ﻿ Dactylorhiza sibirica

Taxon classificationPlantaeAsparagalesOrchidaceae

Efimov

DE6C7C60-2D25-5696-8E4B-64D6B3FF8CED

#### Distribution in adjacent reg.

Russia (Siberia), Kazakhstan (Altai).

#### Specimens examined and literature records.

Eastern Upland: **Pavlodar Region**: Bayanaul District: Bayanaul Mountain Forest, on the slope of the watershed between Jasybai Lake and Sabyndykul, 24 Jun 1979, *Lalayan s.n* (SVER 627698!). **East Kazakhstan Region**: the village of Kriushi, meadow along a stream, 21 Jul 1928, *Ilyin and Heinrichson s.n..* (LE!).

#### Habitat and ecology.

Stream valleys, swampy meadows.

#### Phenology.

Flowering in Jun–Jul; fruiting in Aug–Sep.

#### Conservation status.

Not protected.

#### Notes.

*Dactylorhizasibirica* is reported for the studied region for the first time. This allopolyploid species was described relatively recently, in 2016, with diploid Siberian *D.fuchsii* and *D.incarnata* as its presumable parental taxa (Efimov et al. 2016). In eastern Kazakhstan, the species was earlier incorrectly determined as *Dactylorhizabaltica* (Klinge) Nevski or *Dactylorhiza×kerneri* (Danilova et al. 2020; Sumbembayev et al. 2023).

### ﻿ Dactylorhiza umbrosa

Taxon classificationPlantaeAsparagalesOrchidaceae

(Kar. & Kir.) Nevski

4473B460-9C46-5BAC-B4C2-A324CD8902F9

#### Distribution in adjacent reg.

Russia (Siberia), Kazakhstan (Altai, Western Tien Shan, Balkhash-Alakol Basin, Turanskaya lowlands).

#### Specimens examined and literature records.

Kokchetav: **Akmola Region**: Burabay District: near the Mirnaya Dolina cordon, the shore of Karabulak Stream, 25 Jun 1937, *Sobolev s.n..* (AA!). Tobol-Ishim: **Kostanay Region**: Naurzum District: Nauryzym-Karagay Mountains, 20 May 1909, *Savich and Kucherovskaya 649* (LE!); “Naurzum” State Nature Reserve, 4 Jun 1938, *Siu s.n..* (MW 0816881!). Mugodzhary: **Aktobe Region**: Shalkar District: near Ber-Chogur, 10 Jun 1927, *Rusanov s.n.* (AA!); northwest of Ber-Chogur, on the road to Mugodzharsk, 25 Jun 1927, *Krasheninnikov s.n.*. (AA!). Western Upland: **Karaganda Region**: Zhanaarka District: Sarysu River Valley, “Kara-Agach” place, 13 Jun 1949, *Goloskokov s.n..* (AA!). Eastern Upland: **East Kazakhstan Region**: Ayagoz District: Chingizstau, upper reaches of Kalguta River, 17 Jun 1958, *Gamayunov s.n..* (AA!).

#### Habitat and ecology.

Valleys of rivers and streams, along the damp edges of birch and aspen forests, through swamps, salt marshes, wet meadows, in the lowlands amongst birch thickets.

#### Phenology.

Flowering in Jun–Jul; fruiting in Aug–Sep.

#### Conservation status.

Not protected.

#### Notes.

Aipeisova (2012, 2013) reported *D.majalis*. for Mugodzhary (near Ber-Chogur and Mount Boktybai). Although corresponding herbarium specimens were not located, we believe that this report is an obvious mistake. It is more likely that the plants were *D.umbrosa*, as we found herbarium materials collected from the same place (near Ber-Chogur, 10 Jun 1927, *Rusanov s.n.* (AA!)). We consider *D.umbrosa* and *D.salina* to be closely-related species and determining plants with certainty can sometimes be challenging.

### ﻿ Epipactis atrorubens

Taxon classificationPlantaeAsparagalesOrchidaceae

(Hoffm.) Besser

54835AAF-8718-53BD-8D69-DC3A94AD59E8

#### Distribution in adjacent reg.

Russia (European Russia, Ural, Siberia).

#### Specimens examined and literature records.

Tobol-Ishim: **Kostanay Region**: Uzynkol’skiy District: near Krasnye Borki, 12 Jul 1990, *KSPI students 2189* (LE!, KSPI!).

#### Habitat and ecology.

Pine forests

#### Phenology.

Flowering in Jun–Jul; fruiting in Aug–Sep.

#### Conservation status.

Not protected. The species is very rare and we recommend to include it in the next edition of the Red Book of Kazakhstan.

#### Notes.

For the flora of Kazakhstan, the species was reported relatively recently by Perezhogin et al. (2015), based on the herbarium gathering mentioned above. Field studies are necessary to check whether the plant is still extant at that locality or not.

### ﻿ Epipactis helleborine

Taxon classificationPlantaeAsparagalesOrchidaceae

(L.) Crantz

74F2BC94-2086-5998-97C0-E9703107679E

#### Distribution in adjacent reg.

Russia (European Russia, Ural, Siberia), Kazakhstan (Altai, Western Tien Shan).

#### Specimens examined.

?Mugodzhary: ?**Aktobe Region**: ?Mugalzhar District: Mugodzhar Mts [without detailed locality] (Kusnetsov and Pavlov 1958; Aipeisova 2013).

#### Habitat and ecology.

In mixed and deciduous shady forests, at the forest edges.

#### Phenology.

Flowering in Jun–Jul; fruiting in Aug–Sep.

#### Conservation status.

Not protected. It requires protection at the regional level.

#### Notes.

We did not find any herbarium collections of *E.helleborine* from the studied region and the existing literature reports require confirmation. However, this species is known to be common in mountainous areas of southern and eastern Kazakhstan (Kuznetsov and Pavlov 1958), which are not included in the current revision.

### ﻿ Epipactis palustris

Taxon classificationPlantaeAsparagalesOrchidaceae

(L.) Crantz

857D060E-3F03-5086-9497-8C87CFF47B71

#### Distribution in adjacent reg.

Russia (European Russia, Ural, Siberia), Kazakhstan (Altai, Western Tien Shan).

#### Specimens examined and literature records.

Aktyubinsk: **Aktobe Region**: Uilskiy District: near Uil, 21 Aug 1936, *Nikitin & Deulina s.n.* (LE!). Mugodzhary: **Aktobe Region**: Mugalzhar District: Along the shore of the Shuldak River, 22 Jun 1927, *Rusanov s.n..* (AA!, LE!); Akzerendy River Valley, 4 Jul 1927, *Rusanov s.n..* (AA! LE!); Mugodzhary Mts, the Kunduzdy River Valley (left tributary of the Emba), 20 Jul 1857, *Borszczov 299* (LE!); Shalkar District: Shuldak River Valley (Shet-Irgiz), 29 Jun 1927, *Krashenninikov 638* (LE!). SYRT: **West Kazakhstan Region**: Chingirlauskiy District: “Kara-Agach” place, 13 Jun 1950, *Nikishin s.n..* (LE!). Tobol-Ishim: **Kostanay Region**: Auliekol District: Aman-Karagay pine forest, 10 Sep 1921, *Pavlov s.n..* (LE!); same loc., near Novonezhinka, 3 Jun 1925, *Rusanov s.n..* (LE!); Mendykara District: Borovskaya water protection forest dacha, near Borovskoye, 08 Jul 1925, *Rusanov s.n..* (LE!); Nauruzymsky District: Ak-Kuchuk River Valley, 1 Aug 1929, *Vernander s.n..* (LE!); Kokchetav: **Akmola Region**: Burabay District: Northern swampy shore of Shchuchy Lake, 3 Sep 1981, *Gorchakovskiy s.n..* (SVER 715630!). Irtysh: **Pavlodar Region**: Akkuli District: the shore of Yamyshevskoye Lake, 26 Jun 1913, *Kucherevskaya 1048* (LE!). Semipalatinsk Pinery: **East Kazakhstan Region**: Semipalatinsk District: 25 km east of Semipalatinsk, Kashtak place, 10 Jul 1933, *Sumnevich s.n..* (TK!); same loc., 20 Aug 1933, *Sumnevich s.n..* (TK!) near the Semeytau Mountains, Northern Spring, 25 Jun 1914, *Mordvinova s.n..* (MOSP!); Borodulikha District: Semeytau Mountains, near the farm, 6 Aug 1928, *Zapryagaev 1973* (LE!); Beskaragaysky District: near the mouth of the Shagan River, 23 Sep 1928, *Zapryagaev 2452* (LE!). Karkaraly: **Karaganda Region**: Karkaraly District: Zheltau Mountains (Kupriyanov 2020).

#### Habitat and ecology.

Marshy meadows, river valleys, in wet forests.

#### Phenology.

Flowering in Jun–Jul; fruiting in Aug–Sep.

#### Conservation status.

This species is included in the Red Book of Kazakhstan (category III). It is protected in the following territories: “Naurzum” State Nature Reserve, “Semey Ormany” State Nature Reserve, “Burabay” State National Nature Park, “Karkaraly” State National Nature Park, as well as the State Nature Reserves of “Floodplain of the Irtysh River”, “Orkash”, “Kokzhide-Kumzhargan” and “Budarinskiy”. It is also protected in the natural monument “Birch and pine plantations forests near Borovskoye Lake”. *Epipactispalustris* is one of the most widely distributed orchids in Kazakhstan. Currently, there is a need to reconsider the necessity of state protection for this species.

### ﻿ Epipogium aphyllum

Taxon classificationPlantaeAsparagalesOrchidaceae

Sw.

5EF385DA-958B-5D55-8961-647F653825F9

#### Distribution in adjacent reg.

Russia (European Russia, Ural, Siberia), Kazakhstan (Altai).

#### Specimens examined and literature records.

Karkaraly: **Karaganda Region**: Karkaraly District: Kent Mountains, “Karaagash” place, 4 Aug 1986, *Kupriyanov and Mikhailov s.n..* (KG!).

#### Habitat and ecology.

Swampy pine forests, swamps.

#### Phenology.

Flowering in Jul–Aug; fruiting in Sep–Oct.

#### Conservation status.

This species is included in the Red Book of Kazakhstan (category II) as a rare species found in small numbers within a limited area. In the studied region, it is preserved in the “Karkaraly Biological Reserve”.

#### Notes.

The species in the studied region is known from a single locality in central Kazakhstan, as confirmed by the above herbarium sample. This finding was published in 1987 (Kupriyanov and Mikhailov 1987). Currently, further study is necessary to determine whether this locality is still extant.

### ﻿ Goodyera repens

Taxon classificationPlantaeAsparagalesOrchidaceae

(L.) R.Br.

66297488-7408-5EF8-B086-20DA7086C272

#### Distribution in adjacent reg.

Russia (European Russia, Ural, Siberia), Kazakhstan (Altai, Western Tien Shan).

#### Specimens examined and literature records.

Kokchetav: **Akmola Region**: Burabay District: near Karas’e Lake, 19 Jul 1913, *Semenov s.n..* (TK!); same loc., 10 Aug 1973, *Gorchakovskiy s.n..* (SVER 695775!); same loc., 12 Jun 2011, *Kupriyanov s.n..* (KUZ 00893!); same loc., small southern swamp, 26 May 1973, *Gorchakovskiy s.n..* (SVER 695772!); same loc., big swamp, 29 Jun 1974, *Gorchakovskiy s.n..* (SVER 695770!); Sinyukha Mountain, 17 Jun 1912, *Semenov s.n..* (TK!); same loc., north slope, 24 Jun 1929, *Ilyin s.n..* (LE!); Zolotoborskiy forestry, sq. 24, 18 Jun 1972, *Gorchakovskiy s.n..* (SVER 695773!); Borovsky forest area, near Svetloe Lake, 9 Aug 1973, *Gorchakovskiy s.n..* (SVER 695774!); same loc., near Shortankulskiy peat bog, 5 Aug 1978, *Gorchakovskiy s.n..* (SVER 695776!); Kokchetav Upland, upper reaches of the Imanayevsky Spring, 2 Jul [presumably 1895-1910], *Gordiagin 593* (LE); near the top of Ush-Tas Mount, 2 Jul 1896, *Gordyagin 1105* (LE!); near Burabay, “Burabay” State National Nature Park, Barmashinskoe forestry, 16 Jul 2019, *Kubentayev s.n..* (NUR!).

#### Habitat and ecology.

Moist pine, birch and mixed forests.

#### Phenology.

Flowering in Jul–Aug; fruiting in Sep–Oct.

#### Conservation status.

Not protected. It is necessary to strengthen security measures in the territory of the Shchuchinsko-Borovskaya resort area in the Kokchetav Upland as the habitats of *G.repens* in this area are exposed to strong recreational influences.

#### Notes.

*Goodyerarepens* (Figs 3K, 5F) is found only in a limited area within the Kokchetav FR in the studied region.

### ﻿ Gymnadenia conopsea

Taxon classificationPlantaeAsparagalesOrchidaceae

(L.) R.Br.

AE002BE5-FC77-548C-B28D-5FFD545A7801

#### Distribution in adjacent reg.

Russia (European Russia, Ural, Siberia), Kazakhstan (Altai).

#### Specimens examined and literature records.

Kokchetav: **Akmola Region**: Burabay District: Kokchetav Upland, near Burabay, “Burabay” State National Nature Park, Barmashinskiy forestry, 12 Jul 2019, *Kubentaev s.n..* (NUR!); Kokchetav Upland, at the top of Mount Sinyukha, 22 Jul 1960, *Denisova 1709* (MW 0816897!); Bulandynskiy District: Otradnenskiy forestry, forest quarter no. 121, 4 Aug 1960, *Denisova 2069* (MW 0816896!), same loc., forest quarter no. 11, 4 Aug 1960, *Denisova 1270* (MW 0816895!); Aryk-Balykskiy District: Kokchetav Upland, hills to the south of Imantau Lake, 2 Aug 1960, *Denisova 1817* (MW 0816898!); Zerendi District: 30 km south of Kokshetau, 7 Jul 1957, *Borisova* & *Rachkovskaya 345* (LE!). Tobol-Ishim: **Kostanay Region**: Auliekol District (Auliekol’skiy District): near Aman-Karagay, 27 Apr 1929, *Vernander et al. 331* (LE!). **North Kazakhstan Region**: Kyzylzhar District: right shore of Ishim River, near Krasnoyarka, 29 Jun 1971, *Sologub and Zelinskaya s.n..* (NKU!); same loc., 29 Jun 1971, *Stepanova and Shahvatova s.n..* (NKU!); same loc., 3 Jul 1971, *Troskina and Shandybina s.n.* (NKU!); same loc., Aug 1973, *Wenzler et al. s.n..* (NKU!); same loc., 6 Jul 1974, *Shushakova and Konovalov s.n..* (NKU!); same loc., 06 Jul 1974, *Gorbunova and Mikheeva s.n..* (NKU!); same loc., 9 Jul 1974, *Sinichkina et al. s.n.* (NKU!); same loc., 13 Jul 1974, *Fomenko s.n..* (NKU!); same loc., 14 Jul 1974, *Sokolovskaya s.n..* (NKU!); same loc., 14 Jul 1974, *Spirenkova and Temirbaeva s.n..* (NKU!). Irtysh: **Pavlodar Region**: Shcharbakty District: near Aleksandrovka, 1885, *Golde s.n*. (LE!). Semipalatinsk Pinery: **East Kazakhstan Region** [without detailed locality] (Kusnetsov and Pavlov 1958).

#### Habitat and ecology.

Meadows, birch spikes and grassy pine forests.

#### Phenology.

Flowering in Jun–Jul; fruiting in Aug–Sept.

#### Conservation status.

Not protected. We recommend to include this species in the next edition of the Red Book of Kazakhstan.

#### Notes.

The species was seriously under-recorded in earlier treatments, mainly due to incorrect determinations of the existing herbarium specimens. Thus, no reports are available for Kokchetav and Tobol-Ishim FRs, Kostanay, North Kazakhstan and Akmola administrative regions in the floristic accounts of Kazakhstan (Kusnetsov and Pavlov 1958), Kazakh Upland (Karamysheva and Rachkovskaya 1973; Kupriyanov 2020) and Turgay Depression (Pugachev 1994) for this species. However, there exist older literature reports (Semenov 1928 without detailed localities for Petropavlovskiy Uezd (belongs to Tobolskо-Ishimskiy FR) and Kokchetavskiy Uezd (belongs to Kokchetav FR).

### ﻿ Hammarbya paludosa

Taxon classificationPlantaeAsparagalesOrchidaceae

(L.) Kuntze

C52725B2-9122-5B11-986C-8E9B6E008FB7

#### Distribution in adjacent reg.

Russia (European Russia, Ural, Siberia).

#### Specimens examined and literature records.

Mugodzhary: **Aktobe Region**: Mugalzhar District: Mugodzhary Mts, “Urkach” place, 1 Sep 1934, *Samseev 514* (MW 0816996!).

#### Habitat and ecology.

Sphagnum swamps.

#### Phenology.

Flowering in Jun–Jul; fruiting in Aug–Sep.

#### Conservation status.

Not protected. We recommend to include this species in the next edition of the Red Book of Kazakhstan.

#### Notes.

*Hammarbyapaludosa* was only recently reported for Kazakhstan for the first time (Kubentayev et al. 2021). It was absent in earlier treatments, since the specimen was stored under the name *Microstylismonophyllos* (L.) Lindl. This location is the southernmost part of the area of *H.paludosa.* Fieldwork is necessary to check whether *H.paludosa* is still extant in that locality.

### ﻿ Hemipilia cucullata

Taxon classificationPlantaeAsparagalesOrchidaceae

(L.) Y.Tang, H.Peng & T.Yukawa

14FD558B-60F6-535A-AC72-353B9FA18E75

≡Neottianthecucullata (L.) Schltr. ≡Ponerorchiscucullata (L.) X.H.Jin, Schuit. & W.T.Jin.

#### Distribution in adjacent reg.

Russia (European Russia, Ural, Siberia).

#### Specimens examined and literature records.

Kokchetav: **Akmola Region**: Burabay District: 3 km north of Shuchinsk, 1978, *Grudzinskaya s.n..* (AA!, NUR!); Borovskoy forest area, near Shortankulskiy peat bog, 5 Aug 1978, *Gorchakovskiy s.n..* (SVER!); near Balkashino, in the northern part of the forest dacha of B. Tyukty, 9 Aug 1929, *Grigoriev 367* (LE!); the northern slope of Tuyak-Tau Mountain, 13 Jul 1901, *Gordyagin 529* (LE!); near the Ush-Tas Mount top, 2 Jul 1896, *Gordyagin 1105* (LE!); the eastern shore of Svetloe Lake, 16 Jul 2019, *Kubentaev and Alibekov s.n..* (NUR!); near Burabay, “Burabay” State National Nature Park, Akylbayskoye forestry, 16 Jul 2019, *Kubentayev s.n..* (NUR!); same loc., Borovskoe forestry, 16 Jul 2019, *Kubentayev s.n..* (NUR!); Zerendi District: “Kokshetau” State National Nature Park: Jilandinskoe forestry, near the “Gorodok” cordon, 11 Aug 1960, *Denisova 1973* (MW 0816899!); Ayrtau District: near Shokkaragai, 10 Aug 2020, *Kubentaev et.al. s.n..* (NUR!). Eastern Upland: **Pavlodar Region**: Bayanaul District: Bayanaul Mountains (Gorchakovskiy 1987); same loc., Dzhasybayevsky forestry, 28 Jul 1979, *Lalayan s.n.* (SVER 627695!).

#### Habitat and ecology.

Pine and birch forests.

#### Phenology.

Flowering in Jul–Aug; fruiting in Sep–Oct.

#### Conservation status.

Not protected, but protection is needed since in the studied region the majority of localities fall into the resort area of the Kokchetav Upland. Taking into account that habitats of *H.cucullata* (Figs 3I, 6C) are exposed to serious recreational loads, we consider it necessary to include the species in the next edition of the Red Book of Kazakhstan.

### ﻿ Herminium monorchis

Taxon classificationPlantaeAsparagalesOrchidaceae

(L.) R.Br.

7AA7A4D8-9CBC-52C6-9694-659910CADA4B

#### Distribution in adjacent reg.

Russia (European Russia, Ural, Siberia), Kazakhstan (Altai).

#### Specimens examined and literature records.

Semipalatinsk Pinery: **East Kazakhstan Region**: Semipalatinsk District: near Semipalatinsk, 8 Jun 1914, *Mordvinova s.n..* (MOSP!). Irtysh: **Pavlodar Region** [without detailed locality] (Kuznetsov and Pavlov 1958).

#### Habitat and ecology.

Forest edges, damp meadows.

#### Phenology.

Flowering in Jun–Jul; fruiting in Aug–Sept.

#### Conservation status.

Not protected. We recommend to include this species in the next edition of the Red Book of Kazakhstan.

#### Notes.

*Herminiummonorchis* is reported here for the first time for the Semipalatinsk Pinery FR. Previously, in North Kazakhstan, it was reported only for Irtysh FR (Kuznetsov and Pavlov 1958). In Kazakhstan, *H.monorchis* is more common in the mountainous regions of eastern and southern Kazakhstan, not included in the current revision.

### ﻿ Liparis loeselii

Taxon classificationPlantaeAsparagalesOrchidaceae

(L.) Rich.

8158186E-6194-500F-867E-74E5FBD3459D

#### Distribution in adjacent reg.

Russia (European Russia, Ural, Siberia). Kazakhstan (Balkhash-Alakol Basin).

#### Specimens examined and literature records.

Kokchetav: **Akmola Region**: Burabay District: near Borovoye, the shore of Chebach’ye Lake, 12 Jun 1913, *Drobov 431* (LE!). Semipalatinsk Pinery: **East Kazakhstan Region**: Semipalatinsk District: near Semipalatinsk, 8 Jun 1914, *Mordvinova s.n..* (MOSP!). Mugodzhary: **Aktobe Region**: Mugalzhar District: Mugodzhary Mts, “Urkach” place, 21 Aug 1927, *Krasheninnikov 1230* (LE!).

#### Habitat and ecology.

Sedge and sphagnum swamps.

#### Phenology.

Flowering in Jun–Jul; fruiting in Aug–Sept.

#### Conservation status.

Not protected. The species is extremely rare and we consider it mandatory to include it in the next edition of the Red Book of Kazakhstan.

#### Notes.

*Liparisloeselii* is reported here for the first time for Semipalatinsk Pinery and Mugodzhary FRs. Previously, in the studied region, the species was observed only in Kokchetav FR (Karamysheva and Rachkovskaya 1973; Sultangazina et al. 2014; Kupriyanov 2020). The reported localities of *L.loeselii* belong to the type subspecies, whereas the locality in East Kazakhstan represents the newly-described *L.loeselii* subsp. *Orientalis*, which differs from the typical subspecies by having broader leaf blade, more visible petioles and broader fruits (Efimov 2010).

### ﻿ Malaxis monophyllos

Taxon classificationPlantaeAsparagalesOrchidaceae

(L.) Sw.

78AF6877-8E9C-5F95-907D-A353C6E4DE7E

#### Distribution in adjacent reg.

Russia (European Russia, Ural, Siberia).

#### Specimens examined and literature records.

Tobol-Ishim: **Kostanay Region**: Mendykara District: Borovskaya water protection forest dacha, 17 Jul 1925, *Rusanov 1553* (LE!); near Borovskoye (Pugachev 1994); near Kamenskural’skoe (Pugachev and Masyukova 1969); Auliekol District: near Kalininskoye (Pugachev 1994). Eastern Upland: **Pavlodar Region**: Bayanaul District: Bayanaul Mountains, 30 Jun 1913, *Kucherovskaya s.n..* (LE!); same loc., 10 km west of Bayan-Aul, 19 Jul 1959, *Denisova 436* (LE!); same loc., 19 Jul 1959, *Denisova 427* (MW 0816997!). Karkaraly: **Karaganda Region**: Karkaraly District: Zhisil’tau Mountains, near Egindibulak, 19 Jul 1992, *Kupriyanov s.n..* (KG!).

#### Habitat and ecology.

Grassy birch forests, near lakes, along forest streams.

#### Phenology.

Flowering in Jun–Jul; fruiting in Aug–Sept.

#### Conservation status.

Not protected. We recommend to include this species in the next edition of the Red Book of Kazakhstan.

#### Notes.

Forms both with one and with two well-developed leaves occur in Kazakhstan, the latter being recognised as a variety of M.monophyllosvar.diphyllos (Cham.) Luer (e.g. by Pavlov 1928). Bayan-Aul and Karkaraly Mts represent the southernmost locality of the species in Central Asia.

### ﻿ Neottia camtschatea

Taxon classificationPlantaeAsparagalesOrchidaceae

(L.) Rchb.f.

1041F267-76DC-5B88-A732-0AE63E6AE325

#### Distribution in adjacent reg.

Russia (Siberia), Kazakhstan (Altai, Western Tien Shan).

#### Specimens examined and literature records.

Karkaraly: **Karaganda Region**: Karkaraly District: Kent Mountains, near Kent, 1 Jun 2007, *Kupriyanov et al.* (KUZ 11470!); same loc., “Auletas” place, 25 Jul 1992, *Kupriyanov s.n..* (KG!); Zhisil’tau Mountains, near Egindibulak, 16 Jul 1992, *Mikhailov s.n..* (KG!). Eastern Upland: **Pavlodar Region**: Bayanaul District: Bayanaul Mountains, 10 km west of Bayan-Aul, 19 Jul 1959, *Denisova 444* (MW 0816977!); same loc., 23 Jul 1963, *Denisova 1495* (MW 0816976!).

#### Habitat and ecology.

Damp birch-aspen forests along the shores of streams and lakes.

#### Phenology.

Flowering in Jun–Jul; fruiting in Aug–Sep.

#### Conservation status.

Not protected. The species is extremely rare and we consider it mandatory to include it in the next edition of the Red Book of Kazakhstan.

#### Notes.

*Neottiacamtschatea* is reported here for the first time for Eastern Upland FR and for Pavlodar Region. Earlier occurrences of the species were confirmed in eastern Kazakhstan (Altai, Tarbagatai), southern Kazakhstan (Dzungarian Alatau, Zailiyskiy Alatau) (Kusnetsov and Pavlov 1958) and central Kazakhstan (Karkaraly) (Kupriyanov 2020).

### ﻿ Orchis militaris

Taxon classificationPlantaeAsparagalesOrchidaceae

L.

CC7FA3AF-1CAE-5881-9AF3-929F5A7D8DFB

#### Distribution in adjacent reg.

Russia (European Russia, Ural, Siberia).

#### Specimens examined and literature records.

Prikaspiy: **West Kazakhstan Region**: Chingirlauskiy District: “Kara-Agach”, 23 Jun 1950, *Nikitin s.n..* (LE!); same loc., place valley of the Ural River, headwaters Buldurta River, 21 Jun 2003, *Darbaeva s.n..* (LE!). EMBA: **Aktobe Region**: Mugalzhar District: near Emba, 30 May 1840, *Bunge* 1334 (LE!). Mugodzhary: **Aktobe Region**: Mugodzhary Mts “Urkach” place, near Kumyskul Lake, 10 Jul 1927, *Rusanov 773* (LE!), *s.n..* (AA!); Mugodzhary Mts, near Ayrik (Aipeisova 2012). SYRT: **West Kazakhstan Region**: Bajterekskiy District: south-east of Uralsk, Archiereysky site, s.d. *Gremyachenskiy s.n..* (MW 296812!); Semipalatinsk Pinery: **East Kazakhstan Region**: Semipalatinsk District: near Semipalatinsk, 8 Jun 1914, *Mordvinova s.n..* (MOSP!); Beskaragajskiy District: near Kanonerka, 12 Jun 1996, *Kupriyanov et al. s.n..* (ALTB!). Eastern Upland: **East Kazakhstan Region**: Abaj District: Akshatau Ridge (Karipbaeva et al. 2015).

#### Habitat and ecology.

Sparse birch forests on sandy soils, moist meadows, valleys of rivers and streams, near lakes and forest edges.

#### Phenology.

Northern Kazakhstan: Flowering in Jun-Jul; fruiting in Jul-Aug. Western Kazakhstan: Flowering in May–Jun; fruiting in Jun–Jul.

#### Conservation status.

It is included in the Red Book of Kazakhstan (category III). It is protected in the territory of the following State Nature Reserves: “Semey Ormany”, “Orkash”, “Kokzhide-Kumzhargan”, “Budarinsky”, “Kirsanovsky” and “Ak-Kuma”. In Kazakhstan, *Orchismilitaris* is very rare, the number of individuals in the populations is low. It is necessary to monitor the state of populations.

### ﻿ Platanthera bifolia

Taxon classificationPlantaeAsparagalesOrchidaceae

(L.) Rich.

1471CFF5-72B2-55F7-80FE-48B87441F8D8

#### Distribution in adjacent reg.

Russia (European Russia, Ural, Siberia), Kazakhstan (Altai).

#### Specimens examined and literature records.

Tobol-Ishim: **Kostanay Region**: Mendykara District: Borovskaya water protection forest dacha, 28 Jul 1923, *Rusanov s.n..* (LE!); near Borovskoye, 10 Jul 1977, *Pugachev s.n..* (LE!); botanical nature monument “Plantations of birch and pine forests near Borovskoye Lake”, 24 Jun 2009, *students 195* (KSPI!); Zhitikara District: botanical nature monument “Relict larch-birch grove with Sukachev larch”, 10 Jun 2012, *Perezhogin* (personal observation); Auliekol District: near Kazanbasskoye (Pugachev 1994); near Kalininskoye (Pugachev 1994); near Auliekol (Pugachev and Masyukova 1969). **North Kazakhstan Region**: Kyzylzhar District: right shore of Ishim River, near Krasnoyarka, 30 Jun 1971, *Afonina and Litvinenko s.n..* (NKU!); same loc., 29 Jun 1971, *Stepanova and Shakhvatova s.n..* (NKU!); same loc., 29 Jun 1971, *Sologub and Zelinskaya s.n..* (NKU!); same loc., 28 Jun 1971, *Fesan and Kosmagambetova s.n..* (NKU!); same loc., 29 Jun 1971, *Trushcheleva and Shirokikh s.n..* (NKU!); same loc., 26 Jun 1972, *Kudinova and Schneider s.n..* (NKU!); same loc., 24 Jun 1972, *Makayun s.n..* (NKU!); same loc., 27 Jun 1972, *Rosinskaya and Khrushchev s.n..* (NKU!); same loc., 29 Jun 1972, *Shegebaev and Zhampeisov s.n..* (NKU!). Kokchetav: **North Kazakhstan Region**: Aiyrtau District: near Lobanovo, Kozhevnya swamp, 28 May 2020, *Kubentaev et al. s.n..* (NUR!).

#### Habitat and ecology.

Forest edges and glades, dry meadows, scrub thickets, thinned forests and the outskirts of bogs.

#### Phenology.

Flowering in May–Jul; fruiting in Jul–Aug.

#### Conservation status.

The species is included in the Red Book of Kazakhstan (category III) as an endangered species. It is protected on the territory of the nature monument “Stands of birch and pine forests near Borovskoye Lake”; the botanical nature monument “Relict larch-birch grove with Sukachev larch”; Kokshetau State National Nature Park; and the “Sogrov” State Nature Reserve.

#### Notes.

There is an old report of *P.bifolia* for Kokchetav FR (without detailed locality) by Semenov (1928), which was omitted from the later floristic accounts of Kazakhstan (Kusnetsov and Pavlov 1958) and Kazakh Upland (Karamysheva and Rachkovskaya 1973; Sultangazina et al. 2014; Kupriyanov 2020). Here, we confirm the old data for this floristic region (and simultaneously, for Kazakh Upland) through our recent gathering from the vicinity of Lobanovo, North Kazakhstan Region.

### ﻿ Spiranthes australis

Taxon classificationPlantaeAsparagalesOrchidaceae

(R.Br.) Lindl

64D92CF3-B80F-5524-99B4-A67238C876A5

#### Distribution in adjacent reg.

Russia (European Russia, Ural, Siberia), Kazakhstan (Altai).

#### Specimens examined and literature records.

Tobol-Ishim: **Kostanay Region**: Mendykara District: 4 km south of Borovskoye, 20 Jun 1925, *Rusanov s.n..* (LE!); same loc., 19 Jun 1925, *Rusanov 1490* (MW 0816970!); Auliekol District: near Kalininskoye (Pugachev 1994). Kokchetav: **Akmola Region**: Burabay District: 3 km north of the cordon “Medvezhy”, near Shchuchye Lake, 11 Aug 1973, *Gorchakovskiy s.n..* (SVER 695777!). Irtysh: **Pavlodar Region** [without detailed locality] (Kusnetsov and Pavlov 1958).

#### Habitat and ecology.

On peat bogs.

#### Phenology.

Flowering in Jul–Aug; fruiting in Aug–Sept.

#### Conservation status.

Not protected. Taking into account the limited expansion and the small number of individuals in the population, we recommend to include this species in the next edition of the Red Book of Kazakhstan.

#### Notes.

Currently, field studies are needed to verify the presence of *Spiranthesaustralis* in the study region, since the species was not observed here for almost 30 years.

## Supplementary Material

XML Treatment for Corallorhiza trifida

XML Treatment for Cypripedium calceolus

XML Treatment for Cypripedium guttatum

XML Treatment for Cypripedium macranthos

XML Treatment for Dactylorhiza fuchsii

XML Treatment for Dactylorhiza incarnata

XML Treatment for Dactylorhiza maculata

XML Treatment for Dactylorhiza salina

XML Treatment for Dactylorhiza sibirica

XML Treatment for Dactylorhiza umbrosa

XML Treatment for Epipactis atrorubens

XML Treatment for Epipactis helleborine

XML Treatment for Epipactis palustris

XML Treatment for Epipogium aphyllum

XML Treatment for Goodyera repens

XML Treatment for Gymnadenia conopsea

XML Treatment for Hammarbya paludosa

XML Treatment for Hemipilia cucullata

XML Treatment for Herminium monorchis

XML Treatment for Liparis loeselii

XML Treatment for Malaxis monophyllos

XML Treatment for Neottia camtschatea

XML Treatment for Orchis militaris

XML Treatment for Platanthera bifolia

XML Treatment for Spiranthes australis

## ﻿Additional information

### Author ORCIDs

Serik A. Kubentayev https://orcid.org/0000-0002-0369-0591

Petr G. Efimov https://orcid.org/0000-0003-2926-255X

Daniyar T. Alibekov https://orcid.org/0000-0003-1555-1430

Andrey N. Kupriyanov https://orcid.org/0000-0001-5602-2012

Klara S. Izbastina https://orcid.org/0000-0002-6418-1950

Aizhan E. Khalymbetova https://orcid.org/0000-0002-2584-4766

Yuri V. Perezhogin https://orcid.org/0000-0001-6997-8347

### Data availability

All of the data that support the findings of this study are available in the main text.
